# Decontamination and Germination of Buckwheat Grains upon Treatment with Oxygen Plasma Glow and Afterglow

**DOI:** 10.3390/plants11101366

**Published:** 2022-05-20

**Authors:** Jure Mravlje, Marjana Regvar, Pia Starič, Rok Zaplotnik, Miran Mozetič, Katarina Vogel-Mikuš

**Affiliations:** 1 Department of Biology, Biotechnical Faculty, University of Ljubljana, 1000 Ljubljana, Slovenia; marjana.regvar@bf.uni-lj.si (M.R.); katarina.vogelmikus@bf.uni-lj.si (K.V.-M.); 2 Jožef Stefan Institute, 1000 Ljubljana, Slovenia; pia.staric@ijs.si (P.S.); rok.zaplotnik@ijs.si (R.Z.); miran.mozetic@ijs.si (M.M.)

**Keywords:** cold plasma, seeds, agriculture, fungi, microorganisms, germination, disinfection, plant production

## Abstract

Buckwheat is an alternative crop known for its many beneficial effects on our health. Fungi are an important cause of plant diseases and food spoilage, often posing a threat to humans and animals. This study reports the effects of low-pressure cold plasma treatment on decontamination and germination of common (CB) and Tartary buckwheat (TB) grains. Both plasma glow and afterglow were applied. The glow treatment was more effective in decontamination: initial contamination was reduced to less than 30% in CB and 10% in TB. Fungal diversity was also affected as only a few genera persisted after the glow treatment; however, it also significantly reduced or even ceased the germination capacity of both buckwheat species. Detailed plasma characterisation by optical spectroscopy revealed extensive etching of outer layers as well as cotyledons. Afterglow treatment resulted in a lower reduction of initial fungal contamination (up to 30% in CB and up to 50% in TB) and had less impact on fungal diversity but did not drastically affect germination: 60–75% of grains still germinated even after few minutes of treatment. The vacuum conditions alone did not affect the fungal population or the germination despite an extensive release of water.

## 1. Introduction

Agricultural production has more than tripled since the 1960s. Increased industrialisation and globalisation that have marked the last decades have drastically affected our environment. One of the major characteristics of modern times are dramatically lengthened food supply chains because of the increased urbanisation and changes in our lifestyle. With the rapidly growing human population that is expected to reach almost 10 billion by 2050, the production of food crops should be raised up to 70% to achieve food security [1,2].

Fungal infections are one of the main concerns in cereal grain production that can occur at all stages, from preharvest to postharvest and storage processes [3], whenever temperature and humidity conditions are favourable [4]. Fungi can suppress germination and cause spoilage of the grains [5], reducing their nutritional and organoleptic properties [6]. Some can even produce mycotoxins that are harmful to both humans and animals [7], significantly impacting plant production and food security. As filamentous fungi are ubiquitous, presenting a potential threat to humans and economically important crops, measures are needed to prevent plant material contamination at any stage of production. Fungicides are still the most widely used method to prevent fungal infections of grains [8]. Commercially used pesticides and other artificial chemicals leave residues and negatively impact our environment [9]. Therefore, there is a great interest in finding new, alternative approaches for grain treatment. Cold plasma (CP) treatment techniques seem to offer us potential as a green technology for surface disinfection to prevent fungal growth on seeds and grains [10,11] while avoiding chemical inputs. Besides its antimicrobial activity, CP treatment or treatment with plasma-activated water (PAW) can also stimulate seed germination, enhance growth, and improve the stress tolerance of plants [12,13,14]. Therefore, a new interdisciplinary and rapidly developing field of research known as “plasma agriculture” has been formed in the last few years [15,16]. Some other alternative (non-chemical) approaches also have great potential for microbial inactivation and improving germination of seeds, such as the low-energy electron beam, which is already at the market stage. However, it has been shown that electron beam treatment can have a severe impact on seedling development compared to CP treatment [17].

CP is a partially ionised gas, often referred to as the fourth state of matter. It can be generated by applying thermal or electrical energy to a gas [18]. It consists of free electrons, molecules, atoms, ions, radicals, and other reactive species and represents a source of radiation, in particular, ultraviolet (UV) and vacuum ultraviolet (VUV) photons [19]. All these species give CP unique properties, and although it has a neutral net charge, it conducts electricity [20]. Biological matter such as seeds and grains can be treated with plasma species in two different operational modes: direct (“glow”) or indirect (“afterglow”) exposure. In glow mode, the treated seeds are in direct contact with all species generated by plasma, whereas in afterglow mode, the seeds are placed at a distance from plasma or in an adjacent chamber. This way, they are not exposed to UV or VUV photons or energetic ions, and the amount of plasma active species reaching the seeds is significantly reduced, as only longer-lived plasma species (molecular radicals) affect the treated object [21].

Buckwheat (genus *Fagopyrum*) is a pseudocereal grain crop that belongs to the knotweed family (Polygonaceae) and is a traditional alternative crop grown in Europe and Asia. Although not as widespread and known as some other crops (such as wheat or maize), it is gaining attention as “functional food” exhibiting high nutritional value and numerous beneficial effects on human health. It is rich in proteins, minerals, and phenolic substances, of which flavonoids, primarily rutin and quercetin, predominate [22,23,24,25]. Compared to other crops, a great advantage of buckwheat is that it is gluten-free, and therefore, safe for consumption by patients with celiac disease [26]. Two buckwheat species are predominately cultivated: the widespread common buckwheat (*Fagopyrum esculentum* Moench; CB) and less-known Tartary buckwheat (*Fagopyrum tataricum* Gaert.; TB), grown mainly at higher altitudes in Asia. They are primarily used for flour and groat products to make various foods and traditional dishes [27]. Buckwheat also has quite modest growing demands, making it a suitable crop for organic production [28]. As with most other crops, fungal infections are the number one cause of buckwheat diseases at all stages of production worldwide [29].

Only a few researchers have studied the effects of CP on buckwheat grains. Various CP devices have been tested for their impact on the germination of CB grains [30]. The effects of low-pressure CP treatment on germination and agricultural performance of CB have been presented in [31]; also on germination and fungal community structure of both CB and TB in [32]. Most recently, the effects of plasma-activated water on the growth of TB sprouts were reported [33]. However, up to this point, the effects of different CP treatment modes (glow and afterglow) on germination and decontamination of buckwheat grains have not been studied. This work reports evidence of the effect of glow and afterglow CP treatment on the decontamination of filamentous fungi from both CB and TB. The impact of both treatments on the germination of CB and TB was also studied and explained in terms of plasma characterisation by optical emission spectroscopy.

## 2. Results & Discussion

### 2.1. Plasma Characterisation

Plasma was characterised by optical emission spectroscopy (OES). This method reveals the intensity of radiation from excited atomic and molecular species. The intensity depends on the electron temperature and density, the excitation cross-section, and the concentration of radiating constituents in the original electronic state. The most intensive lines or bands in the optical spectra are usually of resonant origin, and as a result, the relaxation of an excited state by electrical dipole radiation occurs almost immediately after the excitation—the lifetime of resonant states is typically about 1 ns. OES is a qualitative technique for plasma characterisation, and therefore, the comparison of the intensity arising from different radiants is meaningless. Still, the relative intensity provides an insight into the reactions occurring upon treatment of grains with gaseous plasma. The power density of our plasma in H-mode was large (several kW per litre of the volume of dense plasma), and therefore, the influence of the sample on the electron density and temperature was regarded as marginal.

**Figure 1:**
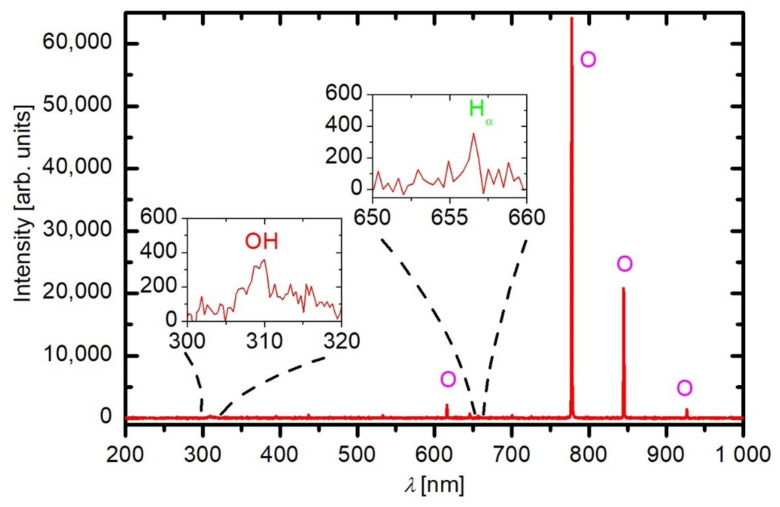
Optical emission spectroscopy of oxygen plasma (50 Pa, 700 W) without any sample (*t*_int_ = 1 ms).

A spectrum of oxygen plasma sustained in the empty discharge chamber is shown in Figure 1. The atomic oxygen lines at 777 and 845 nm dominate the spectrum, which was highly expected because plasma is sustained in oxygen. Although the potential energy of the radiant states was 10.74 and 10.99 eV, respectively, the electrons from the high-energy tail of the distribution function were abundant enough to provide continuous excitation of the resonant levels. There are hardly any other spectral features in Figure 1, and some minor details are highlighted in the inserts. The H-atoms and OH radicals were formed at dissociation of water molecules, and the water molecules in the gas phase appeared because of the residual atmosphere when the samples are absent. Still, the intensity of these spectral features was marginal as compared to the O-atoms lines, indicating the high purity of oxygen in the plasma when the grains are absent.

**Figure 2:**
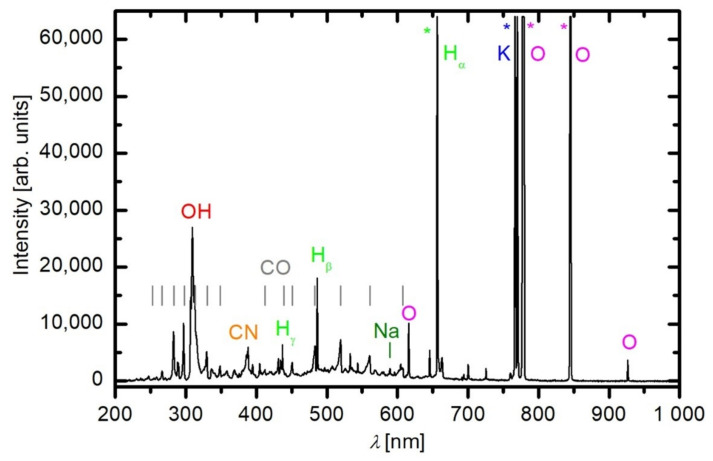
Typical optical emission spectrum during oxygen plasma treatment of grains (*t*_int_ = 100 ms, *t*_treatment_ = 30 s). Grains were treated in the glow of oxygen plasma at 50 Pa and at 700 W. Emission lines in saturation are marked with asterisk.

A typical optical spectrum during the treatment of grains is shown in Figure 2. The most intensive lines were left drawn to saturation in order to make less intensive radiation. The most intensive radiation after 30 s of plasma treatment arose from potassium. This is a macroelement in the plant material, and the reason for such intensive radiation is due to its low excitation energy of the resonant state. Namely, the excited K atoms were relaxed to the ground state, and as a result, the excitation energy of the radiative state was only 1.6 eV. A significant fraction of electrons in gaseous plasma has enough energy for the excitation of the resonant level, and thus K is a significant radiant despite its low concentration in the gas phase. Both oxygen lines at 777 and 845 nm and Hα (Balmer series, transition from the second to the first excited state) were also left to saturation. The high intensity of radiation arising from H-atoms is explained by desorption of water from the grains which were dried in vacuum conditions. The drying, however, did not affect seed germination or fungal spores, which is evident from the results presented below. Some other spectral features are also visible in Figure 2, including the CO and CN bands. The CO bands indicate oxidation of the organic matter. The absence of C2 bands and an extremely faint CH band in the spectrum indicates complete oxidation rather than the thermal desorption of volatile organic molecules in the gas phase.

**Figure 3:**
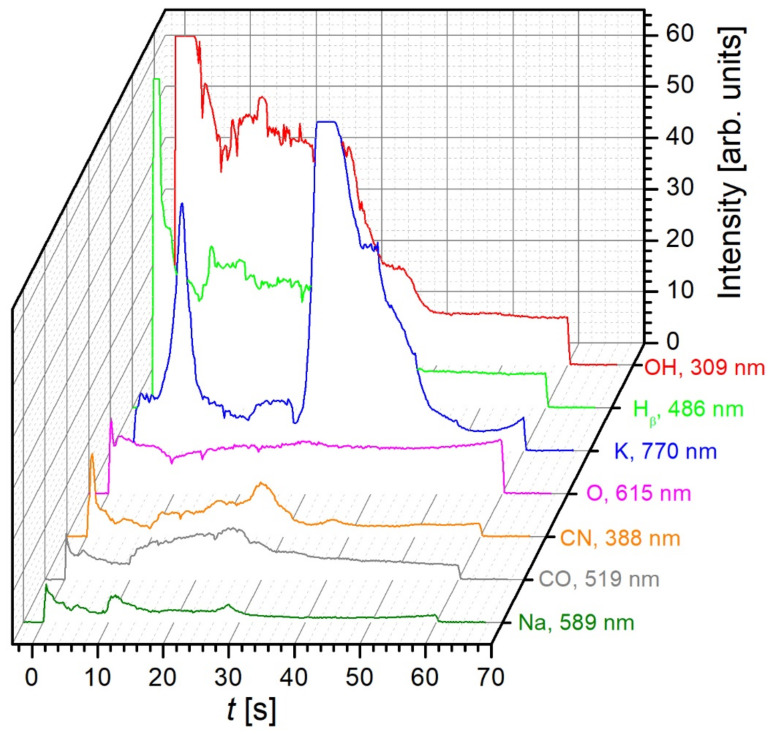
OES intensities of selected spectral features versus the treatment time. Grains were treated in the glow of oxygen plasma at 50 Pa and at 700 W and the OES integration time was 100 ms.

The intensity of the selected spectral features versus the treatment time is shown in Figure 3. Let us first examine the major spectral line of the OH band, the bandhead of 309 nm. The OH band was extremely intensive immediately after the plasma was ignited, due to extensive desorption of water vapour from the sample in the initial stage of plasma treatment. The intensity was large even in the first few milliseconds of plasma treatment when the grains were still at room temperature. Extensive desorption of water was due to the low pressure in the system (well below the saturated water pressure, about 30 mbar at room temperature) and the heating of the sample. As the plasma treatment continued, the intensity of the OH band decreased and dropped below the saturation level already after about 5 s of plasma treatment. Exothermic surface reactions such as neutralisation of charged particles, weak bombardment with positively charged ions, relaxation of metastable species, chemical reactions, and absorption of radiation definitely cause heating of any object placed in plasma, and therefore, the temperature increases with increasing treatment time. Yet, the supply of water vapour from the grains decreased with increasing temperature, somewhat unexpectedly. The paradox is explained by the fact that water boils at low temperatures (well below 100 °C) under vacuum conditions, and therefore, the moisture was desorbed efficiently within the first few seconds of plasma treatment. The OH band persisted to prolonged treatment times (up to a minute), but it may be a combined effect of relatively poor diffusion of water from the interior of grains and oxidation of the organic matter. Similar behaviour was also observed for Hβ, indicating that hydrogen in the gas phase arose from the dissociation of water molecules in the gas phase rather than the decomposition of the organic matter.

The potassium line in Figure 3 exhibited interesting behaviour. Potassium is, of course, chemically bonded in organic matter. Its presence in the gas phase is not a consequence of evaporation (as is the case for water molecules) but indicates chemical etching and/or release of low-mass fragments from the samples. The chemically bonded potassium is then released at dissociative collisions in the gas phase and appears as atomic K. As already mentioned, the excitation level of K lines at 766.5 and 769.9 nm was only around 1.6 eV, and thus the radiation was extensive despite the low density of potassium in the gas phase (or in the organic matter). Immediately after the plasma ignition, the K radiation was poor because the etching was not prominent. The radiation became strong after several seconds (probably due to heating of the grain surface), which may be explained by the presence of K in the pericarp. The K radiation then subsided until about half a minute of plasma treatment, when another extremely intensive peak is observed. The huge radiation intensity arising from K-atoms during 30–40 s of plasma treatment may indicate etching or thermal degradation of cotyledons, which contain the highest amount of potassium and were placed just below the pericarp. Details about the distribution of potassium in the buckwheat grains are reported in [34]. Plasma characterisation, as reported in this paper, may indicate the progression of etching of the grain layers.

The CO band in Figure 3 indicates the chemical etching (oxidation) of organic matter. The intensity of the band was moderate until the first 10 s, and assumed a broad peak in the interval between about 10 and 30 s. The peak may be attributed to the release of low-mass fragments from the surface of the grain. As the temperature increased, the release increased too, but a high temperature suppressed the release of the low-mass fragments, probably due to thermal degradation, which favours carbonisation of the seed surface. The graphite-like materials are oxidised at a much lower rate than the organic matter, and as a result, the intensity of the CO lines (Figure 2 and Figure 3) decreased at longer treatment times. Similar behaviour was also observed for the CN band at the bandhead of about 388 nm. A detailed description of the surface and bulk reactions is beyond the scope of this paper, but the behaviour of the spectral features in Figure 3 gives insight into the process.

### 2.2. Efficacy of Decontamination

**Figure 4:**
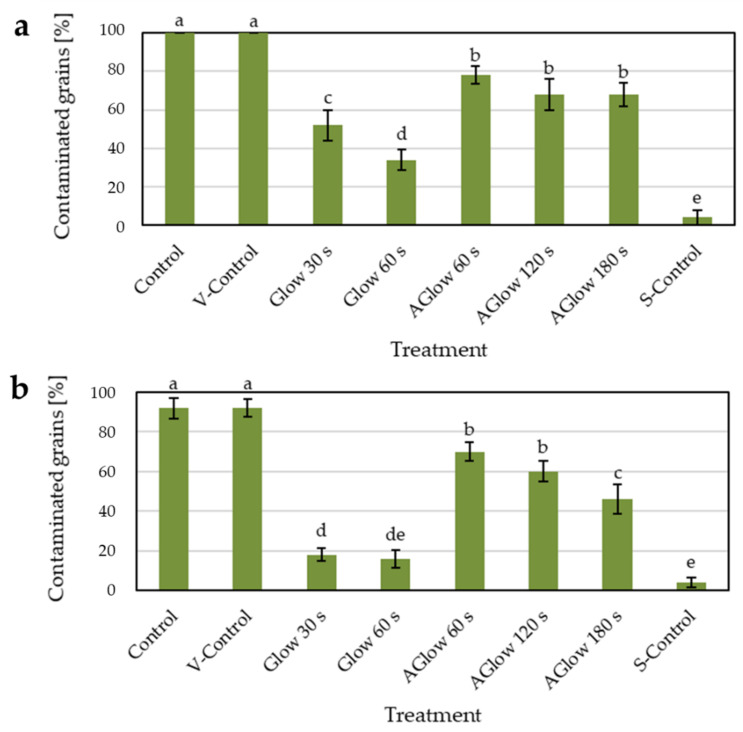
Grain contamination expressed in %. V-Control—vacuum control. S-Control—surface-sterilised control. Aglow—afterglow. (**a**) Common buckwheat; (**b**) Tartary buckwheat. Different letters represent statistically significant differences between groups of grains for each buckwheat species.

Both glow and afterglow CP treatments significantly reduced the percentages of contaminated grains, i.e., the grains from which viable propagules were isolated, in both CB and TB species (Figure 4). The vacuum itself did not affect fungal contamination; therefore, the differences could be attributed solely to the effect of plasma. TB grains had slightly lower initial contamination with fungi (around 90% in both control and vacuum control groups) than CB grains, where all grains were contaminated with fungi in both control groups (100%). This initial contamination probably also affected the efficacy of CP decontamination in both buckwheat species. Glow treatment was found to be significantly more efficient in decontamination than afterglow treatment.

In our experiment, the contamination of CB grains was reduced to less than 50% of the initial value after 30 s glow treatment and less than 35% of initial contamination after 60 s glow treatment (Figure 4). In TB grains, the efficiency of glow CP treatment was even more pronounced, as already after 30 s, less than ¼ of initial contamination was observed, comparable to the 60 s glow treatment in TB. In decontamination efficacy, 60 s glow treatment in TB grains was comparable to surface-sterilised control with 30% H_2_O_2_ (marked as S-Control). This difference in decontamination efficacy between CB and TB could be partially attributed to differences in initial fungal contamination levels, as reported above. Decontamination effects depend not only on the different gas and plasma parameters used but also on the type of contaminated material, especially size and surface shape, which may play a crucial role [6,35]. It remains to be determined whether differences in grain biochemistry can affect decontamination by CP treatments. In our case, grain size and shape were quite different in CB and TB buckwheat, which is further discussed and presented in Section 3.1.

The small low-pressure (LP) CP system used in this study was more efficient in decontamination of buckwheat grains already at shorter treatment times (30 s), compared to our previously reported results with the other, large oxygen radio-frequency (RF) LP CP system, where no significant differences in fungal contamination up to 60 s CP treatment time were observed [32]. That could be attributed to the characteristics of the plasma system, as the CP system used in this study is much more powerful, producing a power density in the glow mode of more than 7000 W L^−1^, compared to the system used in the previous study, which had a power density of only ~30 W L^−1^ [32].

Afterglow treatments also affected the contamination level of buckwheat grains, but not to such a degree as glow CP treatments (Figure 4). There were no significant differences between the 60 s, 120 s, and 180 s afterglow CP treatments in CB grains, whereas in TB grains, contamination was reduced to less than 50% of initial fungal load after 180 s afterglow treatment. The relatively lower decontamination efficacy of afterglow CP treatment can be attributed to the nature of the afterglow mode, as already pointed out. In the glow mode, strong surface etching is present due to higher concentrations of aggressive, reactive species with high energy as well as UV and VUV radiation. In comparison, only longer-lived species with negligible kinetic energy are present in the afterglow mode [36]. In the CP system used, where plasma is generated with an RF generator in an inductively coupled-mode that produces moderately high electron and ion densities, leading to enhancement of surface etching [37], the difference between the two modes was even more evident. Another factor that could affect decontamination is thermal heating. Although reported as minimal degradation effect in the case of pure oxygen, RF-mode generated LP CP as in ours [37], and some authors argue that thermal heating may impact sterilisation efficiency [38]. In the case of afterglow mode, the effect was less pronounced but not negligible, as documented in Section 3. However, the treated grains could heat up significantly in the glow mode due to the extensive exothermic surface reactions, including the absorption of plasma radiation, intense ion bombardment, neutralisation of charged particles, and relaxation of metastables [36,39]. It was observed and measured that the surface of CP-treated samples (hazelnuts) could reach up to 90 °C or even more [40]. However, it was also shown that a reduction in mould contamination occurred due to the detrimental effect of active plasma species, rather than the temperature change during CP treatment, as the viable mould spore count of *Aspergillus* spp. remained the same after 5 min treatment with 100 °C dry heat [40]. That is not surprising, as many moulds that produce survival structures such as conidia and ascospores can survive high temperatures, including some ubiquitous genera common in food and feed, such as *Aspergillus*, *Penicillium*, and *Fusarium* [41]. Unfortunately, our setup did not allow for measuring the temperature of the grain during plasma treatment.

**Figure 5:**
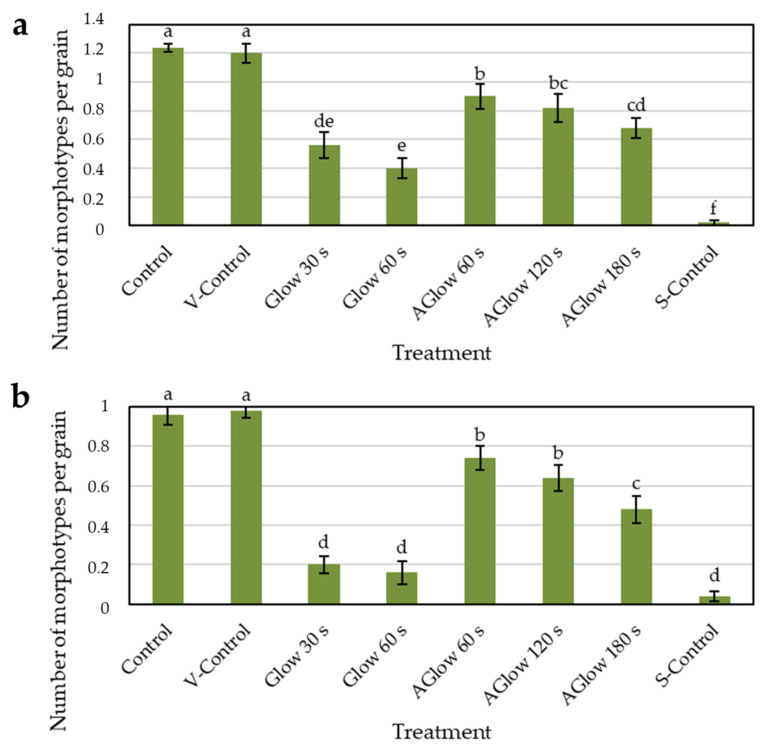
An average number of fungal morphotypes per grain. V-Control—vacuum control. S-Control—surface-sterilised control. Aglow—afterglow. (**a**) Common buckwheat; (**b**) Tartary buckwheat. Different letters represent statistically significant differences between groups of grains for each buckwheat species.

The average number of fungal morphotypes (morphologically different colonies of fungi) per grain decreased significantly after all CP treatments (Figure 5). After both 30 s and 60 s glow treatment, the average number of fungal morphotypes decreased to less than 50% of both control groups in the CB grain. The effect was even more pronounced in the TB grains, where the reduction was comparable to the level of the surface-sterilised grains. Afterglow treatment also significantly reduced the average number of isolated morphotypes per grain in both buckwheat species. Different time exposures to afterglow treatments showed significant differences in the average number of morphotypes per grain in CB between 60 s and 180 s treatments. In TB, the longest (180 s) afterglow treatment significantly differed from the other two afterglow treatments. We already reported a decreasing number of fungal morphotypes with increasing CP treatment in our previous study [32], and therefore, this is in line with our earlier results on the larger oxygen RF LP CP system. The reduction in morphotypes per grain was even more pronounced in this experiment.

### 2.3. Effect of CP Treatment on Fungal Diversity

**Figure 6:**
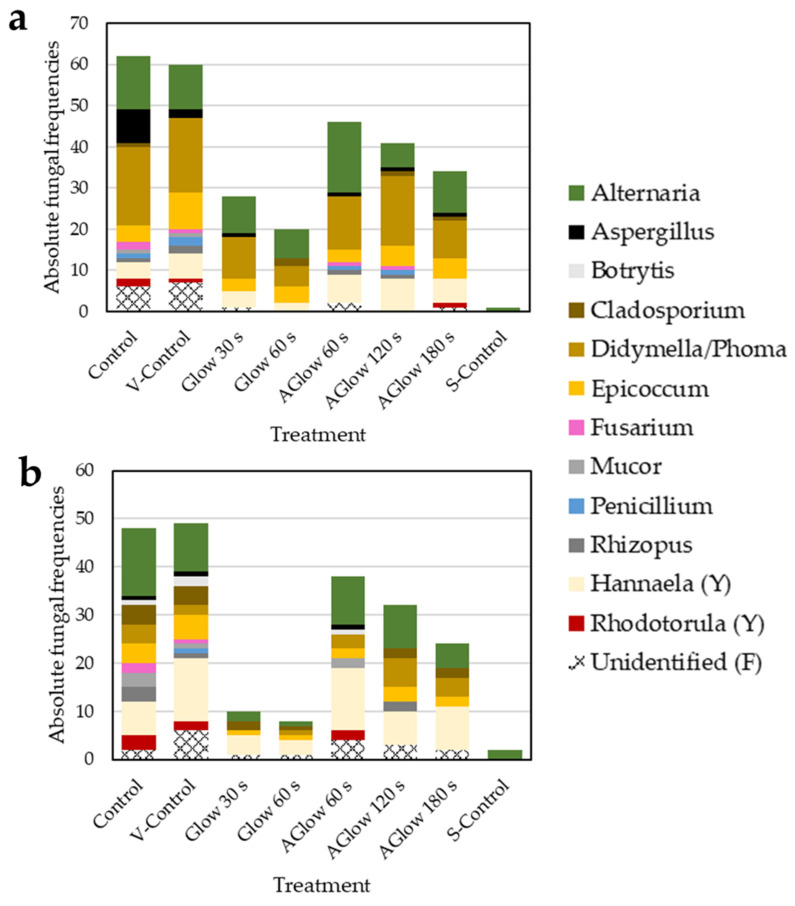
Absolute frequencies and diversity of seed-borne fungi at different treatments. V-Control—vacuum control. S-Control—surface-sterilised control. Aglow—afterglow. (**a**) Common buckwheat; (**b**) Tartary buckwheat. (Y)—yeasts. (F)—filamentous fungi.

Altogether, we isolated 401 fungal colonies (292 from CB and 209 from TB). Isolates were grouped based on their morphological characteristics into distinctive morphotypes. For further morphological identification, supported by molecular identification from our previous studies [32,42], at least two individuals of each morphotype were isolated to pure cultures. After obtaining pure cultures, the morphotypes were attributed to 12 fungal genera. Out of these, ten belonged to filamentous fungi, and two to yeasts. Fungi that could not be unidentified based on their morphology or those we were not able to obtain in pure cultures are listed as unidentified in Figure 6.

In CB grains, genera *Alternaria* and *Didymella* (*Phoma*) prevailed, comprising around 50% of all isolated fungi in both control groups. *Epicoccum* was also a commonly isolated genera, representing 7 and 15% of all isolated fungi in control and vacuum control, respectively. Fungi from genus *Aspergillus* were commonly found in the untreated control group (10% of all isolates). Other filamentous fungi isolated from CB grains that comprised less than 5% of all isolates belonged to genera *Cladosporium*, *Fusarium*, *Penicillium*, *Rhizopus*, and *Mucor*. Yeasts (genera *Hannaela* and *Rhodotorula*) accounted for around 10% of all fungi isolated from CB grains in both control groups. TB grains possessed greater diversity of fungal genera, although total frequencies of fungi were lower than in CB grains (Figure 6 and Table 1). Yeasts were around twice as frequent as CB grains, representing about 20–30% of all isolated fungi in control groups. Among filamentous fungi, species from genera *Alternaria* prevailed, comprising 20 and 29% of all isolated fungi, respectively. *Epiccocum* was also a frequently isolated genus, accounting for roughly 10% of all isolates in both control groups, respectively. Genera *Cladosporium* and *Didymella* (*Phoma*) were also frequently isolated from control grains, representing a little less than 10% of all fungi. As in CB, genera *Aspergillus*, *Fusarium*, *Penicillium*, *Mucor*, and *Rhizopus* were isolated from TB in lower frequencies. Fungi from genera *Botrytis* were found in TB grains but were not present in CB grains. Although the information on seed-borne fungi of buckwheat grains is extremely scarce, similar genera, with *Alternaria* on average being the most common coloniser of buckwheat grains, have been also reported by some other authors [42,43,44,45].

All CP treatments affected the total number of isolated fungi from CB and TB grains and also their diversity (Figure 6, Table 1). With longer CP treatment times, the diversity of fungi found on CB and TB grains decreased, especially in both glow treatments. As in the control group, genera *Alternaria* and *Didymella* (*Phoma*) also prevailed in all CP treatments in CB grains, comprising around 60% of all fungi. Following was genus *Epicoccum*, accounting for about 8–20% of all isolates. Genera *Aspergillus* and *Cladosporium* were isolated from all afterglow treatments, while fungi from genera *Fusarium*, *Penicillium*, and *Rhizopus* were still isolated after 60 and 120 s afterglow CP treatment, but not after 180 s afterglow treatment and both glow CP treatments in CB. Yeasts were relatively evenly represented in all CP treatments (accounting for around 10–20% of all isolated fungi) and their relative frequencies even slightly increased compared to control groups. Similar to CB, in TB grains, genera *Alternaria* and *Didymella* (*Phoma*) prevailed among filamentous fungi after all afterglow treatment, accounting for around 34–50% of all fungal isolates, followed by *Epicoccum*, which represented 6–13% of all fungi. After 60 s afterglow, some other genera of filamentous fungi such as *Aspergillus*, *Botrytis*, and *Mucor* were still isolated from TB grains. After the longest afterglow treatment (180 s), only genus *Cladosporium*, apart from *Alternaria*, *Didymella* (*Phoma*), and *Epicoccum*, was still isolated from TB grains. Yeasts (*Hannaela*) represented up to 40% of all fungi still isolated from TB grains after CP treatments. They presented almost half of all fungi isolated after both glow treatments, which is roughly twice as much as in the control group, suggesting they are more tolerant to CP treatment than filamentous fungi. We already reported in our previous study using a different type of LP CP system for decontamination of buckwheat grains [32] that yeasts were still present after the longest CP treatment. Yeasts and spores are more resistant to CP treatment since they possess extremely thick cell walls made of polysaccharides [46].

In both buckwheat species, 30 s glow CP treatment already significantly affected both absolute frequencies and diversity of fungi, reducing fungal frequencies to less than half of the initial count in CB grains and to almost 1/5 in TB compared to control groups (Figure 3a). This could also be seen from the lowest Shannon diversity indices for 30 s CP treatments presented in Table 1 for both buckwheat species. After 60 s glow CP treatment, the only persisting genera of filamentous fungi in both species of grains were *Alternaria*, *Cladosporium*, *Didymella* (*Phoma*), and *Epicoccum*. Some other authors have also pointed out that *Alternaria* species may be one of the most resistant to CP treatment [47,48,49]. That may prove problematic as this genus is a typical phytopathogen known to cause many plant diseases, such as the black point of cereal species [50]. They are also problematic for buckwheat as they cause poor germination and damping-off [29]. Although they are mainly found as epiphytic fungi and act as plant pathogens, they can also live inside plant tissues as endophytes [51]. *Epicoccum* is a ubiquitous saprophytic fungus, also common as a plant endophyte colonising different tissues in various plants. It is known for producing many secondary metabolites with biological activities and is therefore considered a biological control agent for several plant pathogens [52,53]. Genus *Didymella* (many species formerly known as *Phoma*) is an extremely diverse group of fungi that can act either as plant pathogens or endophytes [51,54,55]. Genus *Cladosporium* is commonly isolated from indoor and outdoor environments, also found as a plant endophyte with plant growth-promoting activity [51,56,57], and a potential source of many natural products for biotechnological and medical applications [58]. All of these genera have already been reported as colonisers of buckwheat grains previously [32,42,43,44].

Our results suggested that glow CP treatment effectively removes fungi that colonise the surface of seeds, i.e., *Aspergillus*, *Penicillium*, and *Mucor*. These fungi are also frequently known as “storage fungi”, infecting seeds and grains during the storage process [59]. These fungi were also less frequently isolated after longer afterglow treatments, indicating that longer afterglow treatments could also effectively decontaminate storage fungi. On the other hand, genera *Alternaria*, *Cladosporium*, *Didymella* (*Phoma*), and *Epicoccum* were found after the longest afterglow and glow CP treatment. This suggests that fungi which can also live as endophytes inside grains may be more resistant to CP treatment. That makes sense as CP treatment acts as a surface decontamination agent and the majority of antimicrobial species have only low penetration depth, making fungi that can live inside plant tissues less susceptible.

### 2.4. Effect of CP Treatment on Grain Germination

**Figure 7:**
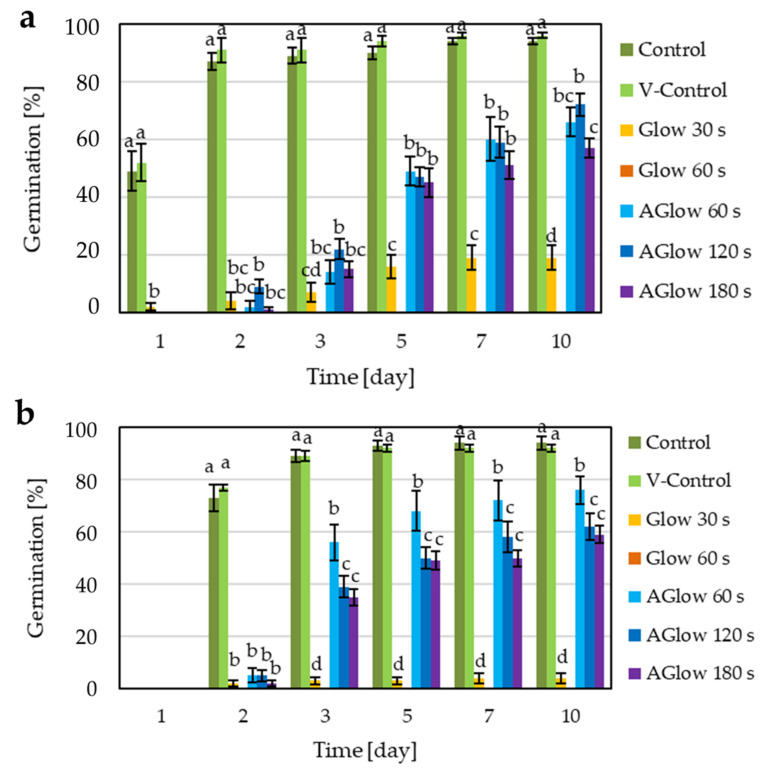
Total grain germination dynamics (as % of all grains) in both buckwheat species on 1st, 2nd, 3rd, 5th, 7th, and 10th day of germination. V-Control—vacuum control. (**a**) Common buckwheat; (**b**) Tartary buckwheat. Different letters represent statistically significant differences between groups of seeds on a specific day in each buckwheat species.

Average germination time for TB grains showed a one-day delay in the germination compared to CB grains. After the second day, the control group of CB grains had already reached around 90% germination and TB grains around 75% (Figure 7). All CP treatments caused a delay in buckwheat grain germination, as shown in Table 2, where mean germination time (MGT) and the time to reach 50% germination (T_50_) were calculated. The same trend was previously reported for buckwheat with RF LP CP system [32].

In all afterglow CP treatments, MGT increased for almost 1 day in TB, whereas in CB, it increased on average for nearly 2 days compared to both control groups. Similarly, T_50_ increased for around 1 day in TB, while in CB, it increased for almost 3 days compared to control groups. However, different time exposures to afterglow treatment had extremely little or no significant effect on both MGT and T_50_ index (Table 2).

Both glow CP treatments had a significant negative impact on germination potential and delayed germination of both buckwheat species. After 30 s glow treatment, the final grain germination was reduced to around 20% in CB grains and to only about 5% in TB grains. These results are in contrast to our previous findings in a different LP CP system [32], where TB grains were somehow more tolerant to CP treatment. That is probably because the small-scale CP system used in this study had around 100-times greater power density (7000 W L^−1^, compared to the power density of about 30 W L^−1^ of the large system). Since TB grains are significantly smaller in size and lighter than CB grains (Table 3), they are probably more susceptible to a powerful CP reactor. As mentioned before, treating samples in a powerful CP reaction can cause heating of the treating object, leading to possible thermal damage. At the physiological level, high temperatures (exceeding 45 °C) are considered lethal because of protein denaturation and degradation and increased fluidity of membrane lipids, which may lead to the death of seed cells and tissues [60]. However, even though the surface temperature of the grains reached or even exceeded 90 °C during CP treatment, the average temperature of the whole grain was assumed to be far below those levels [40]. We also believe that the temperature inside buckwheat grains would be considerably lower than the surface temperature reported in Section 3.2. Regardless of their germination capacity, grains treated in such a way would still theoretically be useful for postharvest applications (storage of foods and feeds) where the detrimental effect of such a CP treatment is not decisive.

On the second day of observation, the germination percentages between different CP treatments (with the exception of 60 s glow treatment, where there was no germination) did not differ significantly. In CB grains, germination percentages between different time exposures to afterglow CP treatments remained comparable up to the 7th day of germination. On the 10th day of observation, the germination of different time exposures to afterglow CP treatments in CB was around 65–70%. In TB grains, the 60 s afterglow CP treatment showed significantly better germination from the third until the last day of observation. On the 10th day, the germination percentage of 60 s afterglow CP treatment reached around 76%, whereas the 120 s and 180 s afterglow CP treatments reached about 60% (Figure 7).

Some authors have studied the effects of various CP treatments on germination and early growth of buckwheat grains. Šera et al. [30] found that plasma had an inhibitory effect on germination and early growth of buckwheat grains and that the effect increased with treatment time. They also reported that the type of plasma and duration of treatment strongly influenced germination and early growth. Ivankov et al. [31] found no difference in the germination of two cultivars of CB in laboratory conditions after CP treatment; however, the percentage of seedlings that emerged under field conditions decreased by 11–20%. Although germination in the field decreased slightly, they found significant improvement in the growth and yield of buckwheats treated with CP. They concluded that pre-treatment of grains with CP stimulated plant growth in the field, and increased biomass production, yield, and nutritional quality. Positive results of plasma-activated water (PAW) on the growth of Tartary buckwheat sprouts were also reported recently [33]. Application of PAW improved germination rate of TB and growth of sprouts increased the content of active substance, and enhanced their antioxidant activity.

This work presents the first evidence on the effects of both glow and afterglow CP treatment on the germination of buckwheat grains. It was shown that the response of wheat seeds to either glow or afterglow treatment may be variety-dependent [61]. There were some differences between two wheat varieties observed, with one being more susceptible to CP treatment than the other. We observed that afterglow treatment caused a more substantial delay in germination in CB than in TB grains, with T_50_ more than doubled. However, the final germination rate of both CB and TB was extremely similar in all afterglow treatments. On the other hand, there was a greater difference in response of the two buckwheat species to glow treatment, with TB being more susceptible. This could be attributed to differences in grain morphology of each buckwheat species, which is shown in Table 3 (Section 3.1). All these factors possibly influenced the results, as the effect of CP treatment on seed germination was already attributed to other factors such as plant species, size and shape of the seeds, seed coat hardiness, and the thickness of the endosperm [62].

## 3. Materials and Methods

### 3.1. Buckwheat Grains Origin and Their Morphological Characteristics

Grains of both buckwheat species were provided by a local Rangus mill (located in Šentjernej, south-eastern Slovenia, at about 230 m a.s.l.). Grains were harvested in 2019 and stored in a dry and dark place at room temperature until the experiments were conducted in 2021.

Grains of different buckwheat species exhibit significant morphological differences (Table 3). Grains of CB are heavier, significantly longer, and especially wider than TB grains, making them rounder than TB grains that are more oblong. TB grains are also lighter, weighing only around 60% of CB grains.

### 3.2. Cold Plasma Treatment

**Figure 8:**
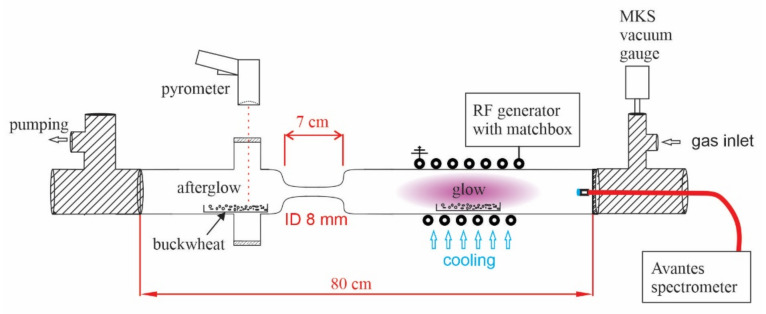
Schematic representation of our home-designed small-scale low-pressure RF plasma system using oxygen as an input gas.

A small-scale low-pressure radio frequency (RF) plasma system schematically shown in Figure 8 was used to treat the buckwheat grains. The system consists of a 4 cm wide glass tube with two segments. The segments are connected with a narrow glass tube to separate the glow from afterglow regions. For each treatment, 100 grains were spread on a metallic mesh. For the treatment of grains in a glow (direct) plasma regime, the mesh was placed in the copper coil area of the tube. For the treatment of grains in the afterglow (indirect) plasma regime, the metallic mesh was placed in the tube outside of the coil area, right after the narrowing part of the tube, as illustrated in Figure 8. Oxygen gas of 99.999% purity was supplied to the plasma reactor at a constant flow rate of 60 sccm. Plasma was generated at low-pressure conditions (50 Pa) and RF power of 700 W. The RF generator operated at the frequency of 13.56 MHz to which the copper coil was connected. The power density was around 7000 W L^−1^. We treated the grains in the glow plasma region for 30 and 60 s and the afterglow region for 60, 120, and 180 s. Right after the plasma treatment, the grains were vacuum-packed in sterile PVC bags to prevent contamination from the environment.

The temperature of the grains in the afterglow chamber was estimated with an infrared (IR) pyrometer as shown in Figure 8. The pyrometer was focused on the grains (the focal spot diameter was about 7 mm), and an IR-transparent vacuum-tight window was mounted between the grains and the pyrometer. The temperature of grains was slowly increased with the afterglow treatment time and reached about 60 °C after a minute. The temperature was almost 100 °C after 3 min of afterglow treatment. Here, it is worth mentioning that the IR pyrometer may not be an extremely accurate technique for measuring the sample temperature because of the unknown variation of the emissivity in the IR range due to modifications in the surface properties.

Furthermore, it should be stressed that the pyrometer estimates the temperature of the grain surface. The temperature inside the grain is definitely lower, but it is not trivial to measure it. Theoretically, it is possible to calculate the evolution of the temperature inside the grains, but a complex differential equation should be solved. Such calculations are beyond the scope of this article, especially as the coefficients for thermal conductivity and capacity have been only estimated for a limited number of seeds, for example, lentils [63].

The temperature of the grains should be greater in the case of direct treatment in the glowing plasma. Unfortunately, it was not feasible to measure the surface temperature of the grains within the coil since the measurements would require an IR-transparent window which is mounted between the coil turns. The exothermic reactions in the glowing plasma include the heterogeneous surface recombination of oxygen atoms, relaxation of metastables, weak bombardment with positively charged ions (the samples are always at the floating potential in glowing plasma), neutralisation of charged particles, and absorption of light quanta. The heat dissipated on the surface of the grains could be calculated if the fluxes of all these plasma species were known. Unfortunately, the system shown in Figure 8 is not equipped with instruments for measuring these plasma parameters. Furthermore, the coefficients were not known for these types of samples.

Optical spectra were measured with a calibrated spectrometer (AvaSpec-3648 Fiber Optic Spectrometer, Avantes, Apeldoorn, The Netherlands) of a wavelength span between 200 and 1000 nm. The integration time of the spectrometer was 1 ms. The rotational temperature of OH radicals was estimated from the main peak at about 310 nm using LIFBASE 2.1 software. LIFBASE is a free software program to chart the spectroscopy of diatomic molecules. The best fit was obtained at the rotational temperature of OH radicals of about 2000 K. The rotational temperature indicates the kinetic temperature, providing the coupling of rotational and translational states is optimal.

### 3.3. Decontamination Test

For evaluating decontamination efficiency, the grains from all CP treatments (glow and afterglow), untreated grains (positive control), and chemically surface-sterilised grains (with 30% H_2_O_2_ for 20 min; negative control) were used and analysed as described below.

#### 3.3.1. Cultivation of Seed-Borne Fungi

For the cultivation of seed-borne fungi, the direct agar plate or “Ulster” method was used to evaluate grain colonisation and fungal growth. Sterile plastic Petri dishes (diameter 90 mm) with 2% potato dextrose agar (PDA) supplemented with the antibiotic chloramphenicol (50 mg L-1) were used for the cultivation of fungi. For each group of grains, five buckwheat grains were placed in a circle evenly distributed around 2 cm from the edge of the Petri dish so that the fungi could grow around each grain. Plates were incubated in growth chambers at a temperature of 22 °C in dark conditions for one week.

#### 3.3.2. Degree of Colonisation and Morphological Identification of Fungi

After a week of cultivation, we examined the plates. The degree of contamination was measured as a percentage of colonised grains. The diversity of fungi around each grain was evaluated as the number of different fungal colonies (morphotypes) grown from each grain. Based on their appearance, all grown fungi were visually examined and grouped by morphotypes (morphologically distinctive fungal colonies). At least two individuals of each morphotype were isolated in pure cultures for further identification. Based on macroscopic and microscopic observations [64,65] and our previous fungal isolates from both CB and TB grains confirmed with molecular identification [32,42], our isolates were identified as 12 different fungal genera. Fungi that we were not able to isolate in pure cultures or could not identify based on their morphology are presented as “unidentified”. The percentage of colonised grains (CG) and the average number of morphotypes per grain (MG) were calculated according to Equations (1) and (2), respectively:

(1) CG (%)=(number of colonised grains)/(total number of grains)×100

(2) MG=(∑number of morphotypes per plate)/(total number of morphotypes)

The diversity of fungal genera in each group of grains was assessed using a Shannon diversity index, calculated as:

(3) $$\mathrm{Shannon}\;\mathrm{Diversity}\;\mathrm{Index}\;(H)=-\overset{s}{\underset{i=1}{\sum }}p_{i}\;\ln\;\left(p_{i}\right)$$

### 3.4. Germination Tests and Indices

In germination tests, 20 grains from each CP treatment (glow and afterglow), vacuum control grains, and untreated grains were placed into Petri dishes (diameter 70 mm) with two layers of filter paper and moistened with 3 mL of sterile distilled water. For each group of grains, the germination test was performed in five replicates. The grains were incubated in plant growth chambers at 22 °C, with 60% humidity, in the dark. After five days of incubation, a little bit of sterile distilled water was added to the Petri dishes to maintain constant humidity during germination. The number of germinated grains was counted every 24 h for the first three days and then on the 5th, 7th, and 10th day. The germination rate (GR%) was calculated according to Equation (4). The visible penetration of the radicle through the seed coat was used as the criterion for germination.

(4) GR (%)=(number of germinated grains)/(total number of grains)×100

To better evaluate germination process, mean germination time (MGT) was calculated according to the equation of Ellis and Roberts (1981) [66]:

MGT = ΣD × n/Σn(5)

where n is the number of seeds germinated on day D, and D is the number of days counted from the beginning of germination.

Besides MGT, the time to 50% germination (T_50_) was also calculated according to the equation of Coolbear et al. (1984) modified by Farooq et al. (2005) [67] as:

T_50_ = t_i_ + [(N/2 − n_i_) (t_i_ − t_j_)]/(n_i_ − n_j_) (6)

where N is the final number of germination and n_i_, n_j_ is the cumulative number of grains germinated by adjacent counts at times t_i_ and t_j_ when n_i_ < N/2 < n_j_.

### 3.5. Grain Characteristics

Morphological characteristics (length, width, and weight) of both CB and TB grains were also evaluated. Length and width (maximum width) were measured using a digital Vernier caliper. Fifteen randomly selected grains of each buckwheat species were measured. For weight measurement, ten grains of each buckwheat species were weighed using digital scales (in 5 replicants), and the average mass per seed was calculated.

### 3.6. Statistical Analysis

All results reported above are expressed as mean ± standard error (SE) of ten replicates (for decontamination) or five replicates (for germination tests). Statistical significance between groups of grains (different treatments) was determined using one-way analysis of variance (ANOVA) with Duncan’s post hoc test (using Statistica StatSoft version 7). The significance level was considered at a p-value of less than 0.05.

## 4. Conclusions

Searching for modern and alternative techniques to eliminate fungal contamination in agricultural production and the food industry is essential to reducing the use of fungicides and other toxic chemicals. Cold plasma treatment of seeds and grains is a “green technology” that can be utilised as an economically sustainable and environmentally friendly alternative to help us solve some of the greatest challenges of modern times, such as sustainable agriculture and food security for the ever-growing human population. The method was employed for the treatment of buckwheat grains. Plasma was characterised by optical emission spectroscopy (OES) to obtain some insight into the surface reactions upon treatment with glowing plasma. The interaction between the plasma species and grains was extensive. The presence of the grains inside the plasma reactor caused a significant release of water vapour. Water molecules efficiently dissociated to H and OH radicals, which, apart from O-atoms, prevailed among chemically reactive species, which caused significant etching. The temporal evolution of the most significant spectral features indicated that OES may be a rather powerful and simple technique for monitoring the etching evolution. Of particular interest was the behaviour of the radiation arising from resonant potassium states. Considering the distribution of this element in the grain constituents, one may be able to follow the etching progression.

With the results presented in this paper, the effects of glow and afterglow low-pressure CP treatment on decontamination and germination of both CB and TB grains could be compared. We showed that glow treatments were extremely effective in the decontamination of both buckwheat grains. The contamination was reduced to less than 30% of initial contamination in CB, and less than 10% in TB. Glow treatment also strongly affected fungal diversity, with genera *Alternaria*, *Cladosporium*, *Didymella* (*Phoma*), and *Epicoccum* recognised as the most resistant to CP treatment. However, the treatment probably would not be the best choice to treat buckwheat grains for presowing application as germination was drastically reduced after 30 s CP treatment or even ceased after 60 s CP treatment. Instead, it can be utilised as a postharvest treatment for food and feed (where grains no longer have to germinate) if the compound and nutritional composition of the grains remain intact. This is something that would be valuable for further investigations and research.

On the other hand, afterglow treatments showed less efficacy in decontamination, as the overall contamination was reduced on average by around 20–30% compared to control. Nevertheless, the longest (180 s) afterglow CP treatment in TB grains reduced initial contamination to less than 50%. Afterglow treatment also did not affect fungal diversity as much as glow treatments. Nonetheless, it had a substantially less negative effect on the germination of both CB and TB grains. From this perspective, in a powerful plasma setup such as the one used in our study, afterglow (indirect) treatment is more appropriate for treating buckwheat grains than glow (direct) treatment, as long as the germination ability is the main goal.

## Figures and Tables

**Table 1:** Shannon diversity index calculated for each group for both buckwheat species.

Treatment	Common Buckwheat	Tartary Buckwheat
Control	1.886	2.122
Vacuum Control	1.852	1.940
Glow 30 s	1.383	1.273
Glow 60 s	1.496	1.475
Afterglow 60 s	1.547	1.688
Afterglow 120 s	1.675	1.636
Afterglow 180 s	1.630	1.448
Sterilised Control	0	0

Note: unidentified fungi that were almost randomly and evenly distributed through all groups were excluded from the calculation.

**Table 2:** Mean germination time (MGT) and time to reach 50% germination (T_50_) were calculated for both buckwheat species for both control groups and all CP treatments. *n.d.* indicates that indices could not be calculated due to extremely low germination (less than 20%). Different letters (superscripts) indicate statistically significant differences between groups of grains per each buckwheat species.

	CB		TB	
Treatment	MGT	T_50_	MGT	T_50_
Control	4.87 ^a^	1.01 ^a^	5.38 ^a^	1.65 ^a^
Vacuum C.	4.84 ^a^	0.96 ^a^	5.32 ^a^	1.60 ^a^
Glow 30 s	*n.d.*	*n.d.*	*n.d.*	*n.d.*
Glow 60 s	*n.d.*	*n.d.*	*n.d.*	*n.d.*
AGlow 60 s	6.84 ^c^	4.05 ^b^	6.18 ^b^	2.67 ^b^
AGlow 120 s	6.59 ^b^	4.12 ^b^	6.27 ^b^	2.77 ^bc^
AGlow 180 s	6.75 ^bc^	3.88 ^b^	6.33 ^b^	2.92 ^c^

**Table 3:** Morphological characteristics (weight, length, and maximum width) of both buckwheat grains. Different letters (superscripts) indicate statistically significant differences between common buckwheat and Tartary buckwheat grains.

Parameter	Common Buckwheat	Tartary Buckwheat
Weight (mg)	25.9 ± 0.9 ^a^	16.3 ± 0.8 ^b^
Length (mm)	5.33 ± 0.1 ^a^	4.96 ± 0.1 ^b^
Width (mm)	3.87 ± 0.0 ^a^	2.75 ± 0.1 ^b^

## Data Availability

All data presented in this study are available in this paper.
